# Evaluation of Mechanical and Shrinkage Behavior of Lowered Temperatures Cementitious Mortars Mixed with Nitrite–Nitrate Based Accelerator

**DOI:** 10.3390/ma13173686

**Published:** 2020-08-20

**Authors:** Yusuke Tomita, Akira Yoneyama, Heesup Choi, Masumi Inoue, Jihoon Kim, Hyeonggil Choi, Yuhji Sudoh

**Affiliations:** 1 Department of Civil and Environmental Engineering, Kitami Institute of Technology, Hokkaido 090-8507, Japan; m2052200130@std.kitami-it.ac.jp (Y.T.); m1952200234@std.kitami-it.ac.jp (A.Y.); m-inoue@mail.kitami-it.ac.jp (M.I.); 2 Faculty of Environmental Technology, Muroran Institute of Technology, Hokkaido 090-8585, Japan; bmjhun@mmm.muroran-it.ac.jp; 3 School of Architecture, Civil, Environment, and Energy Engineering, Kyungpook National University, Daegu 41566, Korea; hgchoi@knu.ac.kr; 4 Basic Chemicals Department Chemicals Division, Nissan Chemical Corporation, Tokyo 103-6119, Japan; sudouyuuji@nissanchem.co.jp

**Keywords:** frost-resistant accelerator, calcium nitrite, calcium nitrate, cracking, strength, pore volume, shrinkage, crack potential, degree of restraint

## Abstract

Recently, calcium nitrite (Ca(NO_2_)_2_) and calcium nitrate (Ca(NO_3_)_2_) have been increasingly used as the main components of salt- and alkali-free anti-freezing agents, for promoting concrete hydration in cold-weather concreting. With an increase in the amount of nitrite-based accelerator, the hydration of C_3_A, C_3_S, and βC_2_S in the cement is accelerated, thereby improving its early strength and effectively preventing the initial frost damage. Meanwhile, with an increase in the amount of nitrite-based accelerator, the expansion and shrinkage of the concrete—and, therefore, the crack occurrence—are expected to increase. In this study, various experiments were conducted on shrinkage, crack initiation, and the development of mortar containing a considerable amount of a nitrite-based accelerator. The result confirmed that, as the amount of nitrite-based accelerator was increased, the shrinkage was increased, and cracking in early age was more likely to occur, compared to the cases without the addition of this accelerator.

## 1. Introduction

During cold-weather concreting, to prevent the initial frost damage, it is necessary to control the temperature until the concrete strength reaches 5 N/mm^2^ of the heat curing, using a temporary enclosure and heater [1,2,3,4,5,6]. On the other hand, under a very low temperature or worse conditions—such as a steep incline, narrow space, or windstorm—anti-freezing agents are used to prevent the initial frost damage and secure the initial strength by sheet curing. Generally, the allowable range of anti-freezing agents is from −4 to −8 °C [7,8,9,10]. However, when the daily average temperature is below −10 °C, it is necessary to increase the amount of anti-freezing agent.

At present, calcium nitrite and calcium nitrate are used as the main components of a salt- and alkali-free-type nitrite-based accelerator (CN) [9,10]. It has been proposed that increasing the amount of this nitrite-based accelerator contributes to good initial strength development in low-temperature environments, due to the hydration promotion of C_3_A in the cement—and that is due to the increased solubility of C_3_S and βC_2_S [11,12,13]. According to the Japanese Architectural Standard Specification for Reinforced Concrete Work (JASS5), shrinkage cracks need to be reduced to ensure the durability of concrete. Therefore, the drying shrinkage rate of concrete has been stipulated to be under 8 × 10^−4^ [14].

CN accelerates the hydration reaction of the C_3_A contained in cement and increases the amount of ettringite and monosulfate [15,16,17]. This is because hydrates, such as nitrite–nitrate hydrate (3CaO·Al_2_O_3_·Ca (NO_2_/NO_3_)_2_·xH_2_O), are formed through reactions between the C_3_A (Al_2_O_3_), NO_2_^−^, and NO_3_^−^ in CN [15,16,17]. Therefore, the chemical shrinkage potential of the cement matrix increases, which leads to a higher concern regarding shrinkage cracking. However, few studies have focused on shrinkage cracking.

**Figure 1:**
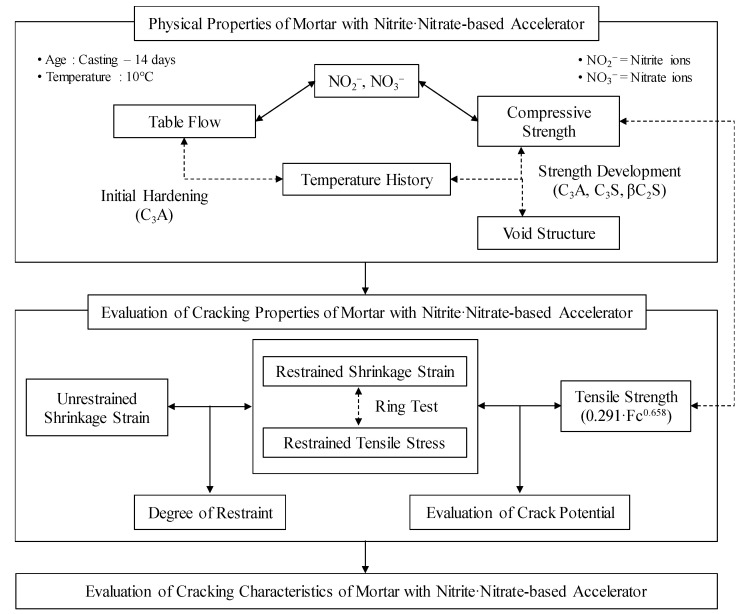
Study flow chart.

In this study, the authors’ aims are to clarify the shrinkage behavior of concrete, and the initiation and development of cracks, when a considerable amount of CN is present in the mixture. Therefore, various experiments have been conducted to quantitatively evaluate the physical and shrinkage cracking properties of mortar containing a considerable amount of CN. Figure 1 shows the flowchart for this study.

## 2. Experimental Overview

### 2.1. Materials and Procedures

Table 1 presents the materials used in the experiments conducted in this study, and Table 2 presents their CN components. CN is a 45% mixed aqueous solution of calcium nitrite and calcium nitrate. Table 3 presents the mortar composition used in the experiments, where the water–cement ratio is 50% and the sand–cement ratio (S/C) is 2.5 [10,11]. The standard amount of accelerator added is 4%~7% of the cement mass, depending on the ambient temperature [10]. Assuming a case where a considerable amount of CN was added in extremely low temperatures, the amount of CN added was set to the four levels of 0, 7, 9, and 11%, as compared to the cement weight. The concrete temperature during the unloading has been specified as 10~20 °C by the Architectural Institute of Japan in the Recommendation for Practice of Cold Weather Concreting (Practical Guideline for Investigation (2010)). Therefore, in the experiment, to elucidate the behavioral assessment of the concrete expansion and shrinkage when a CN is added, both mixing and sealed curing were performed at 10 ± 1 °C and 85% ± 5% (Relative Humidity; RH), from Day 1 to Day 14, after placing.

### 2.2. Experimental Method

As the amount of nitrite-based accelerator was increased, various physical and shrinkage behavior assessments were conducted at various ages, using the experimental method outlined below. Table 4 shows the conditions and assessment method applied in this experiment.

The mortar’s fresh property was according to the “Flow test” in JIS R 5201 (Physical Testing Methods for Cement; Tokyo, Japan), and a flow test was performed immediately after mixing [19].

To assess the compressive strength, sealed curing was performed at 10 ± 1 °C and 85% ± 5% RH, from Day 1 to Day 14, after placing the mortar into a φ 5 × 10 cm^2^ mold. Next, the compressive strength was measured on Days 1, 3, 7, and 14.

For the internal temperature, a thermocouple (Tokyo, Japan) was installed at the center of a mold of 10 × 20 cm^2^, and the time-dependent change in the mortar temperature immediately after mixing, until Day 14, was measured.

**Figure 2:**
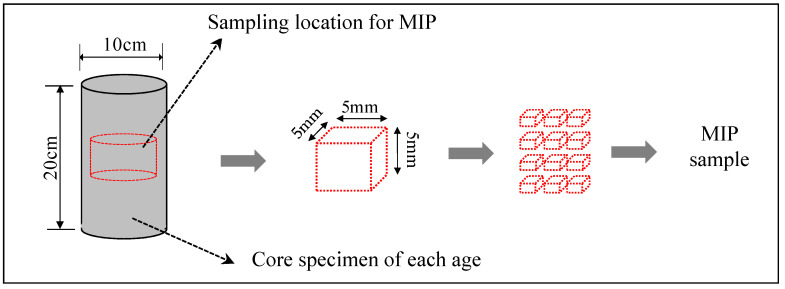
Sampling of specimen for evaluation of pore structure.

For the MIP (Mercury Intrusion Porosimetry) test, as shown in Figure 2, in order to obtain a sample that is representative of the measurement of porosity, samples were collected at each age and cut into 5 mm cubes. Then, in order to stop the hydration of the sample, the sample was immersed in acetone for 4 h or more, and dried by the D-dry method for 1 week, and then the pore distribution of each sample was measured at a minimum diameter of 6 nm, with a maximum pressure of 220 MPa.

**Figure 3:**
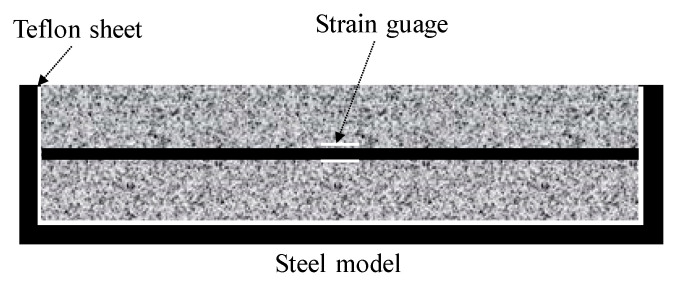
Overview of unrestrained drying shrinkage.

As shown in Figure 3, the unrestrained drying shrinkage strain was measured by embedding a reinforcing bar with a strain gauge that was attached to the center of a 10 × 10 × 40 cm^3^ mold. To make the drying conditions identical to those of the ring specimen, and after demolding the specimen at the age of 1 day, the exposed surface area ratio was adjusted to be the same as that of the ring specimen (~44%) by covering the circumferential direction with aluminum tape.

**Figure 4:**
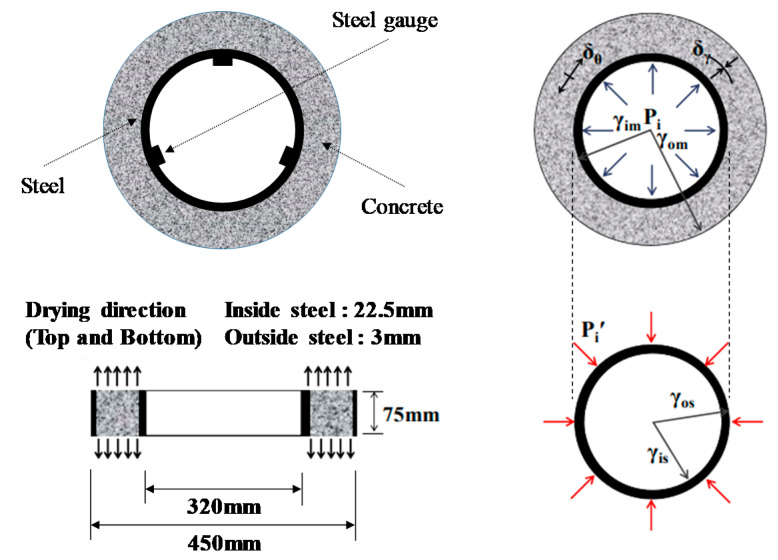
Overview of ring test (restrained shrinkage).

**Figure 5:**
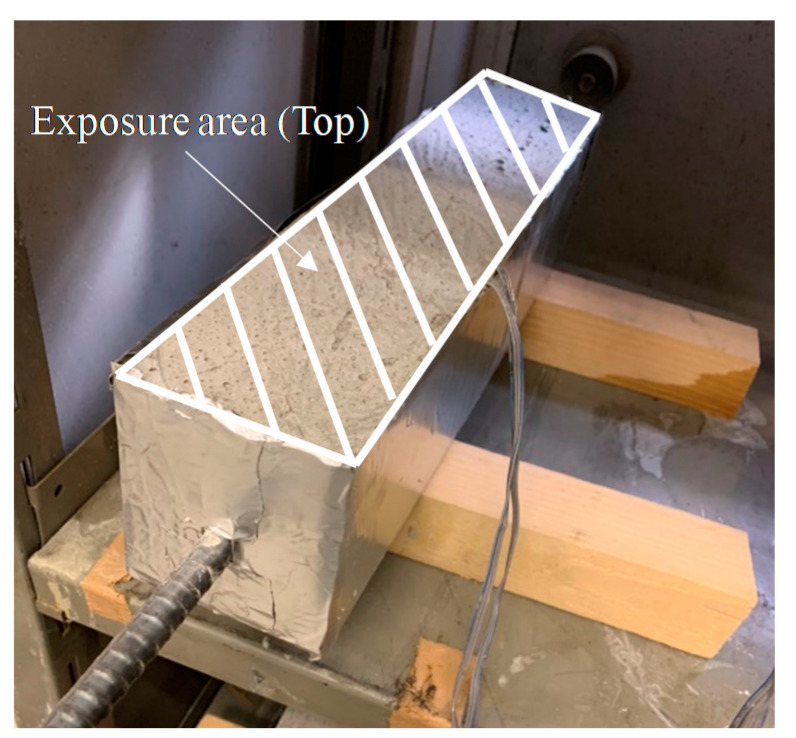
Unrestrained drying shrinkage.

**Figure 6:**
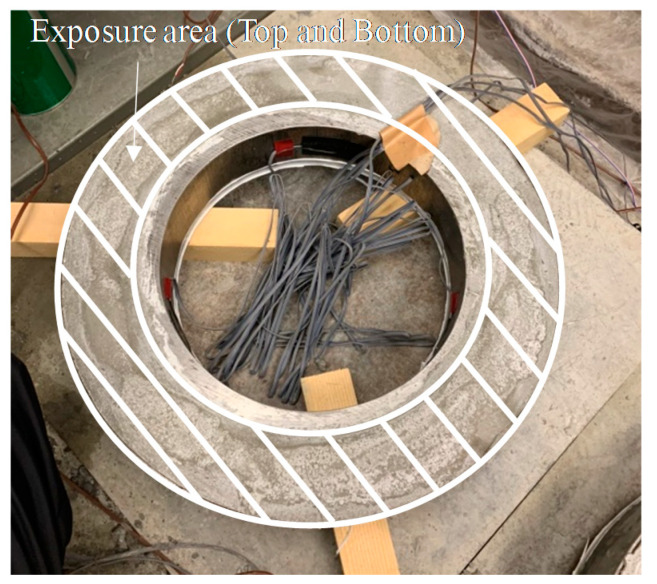
Restrained shrinkage (ring test).

The restraint shrinkage strain is a strain generated by the restraint of the inner steel ring. The height of the ring test piece was changed from 152 to 75 mm, proposed by AASHTO (American Association of State Highway Transportation Officials; 1998), to induce uniform dry shrinkage at the cross-section of the concrete ring (Figure 4) [20,21]. In this experiment, a Teflon sheet was installed between the outer ring and the mortar to minimize the restraint from the outer ring and prevent water evaporation on the upper part of the test body. At the age of 1 day, the lower wood plate and upper Teflon sheet of the ring specimen were removed, and thus, only the upper and lower surfaces were dried. For determining the restrained shrinkage strain, strain gauges were attached at three locations (h/2 = 37.5 mm) in the center of the inner ring, and the change in the strain with time was measured immediately after that. Figure 5 shows the apparatus of the unrestrained drying shrinkage test, with the specimen’s exposed surface area ratio adjusted to be the same as that of the ring specimen. Figure 6 shows the apparatus of the restrained shrinkage test (ring test).

## 3. Physical Properties of Mortar with CN

### 3.1. Fresh Properties

**Figure 7:**
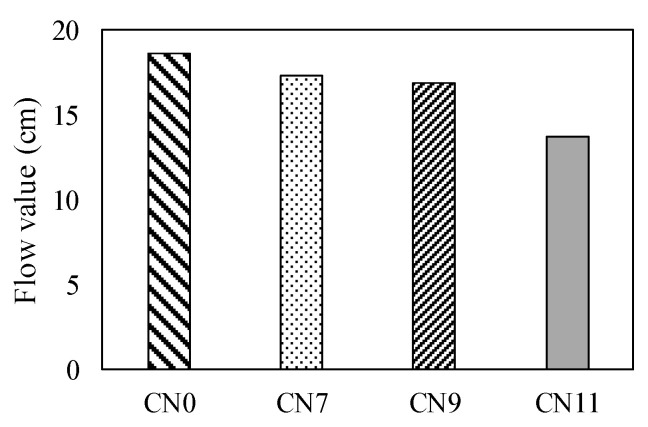
Flow value.

**Figure 8:**
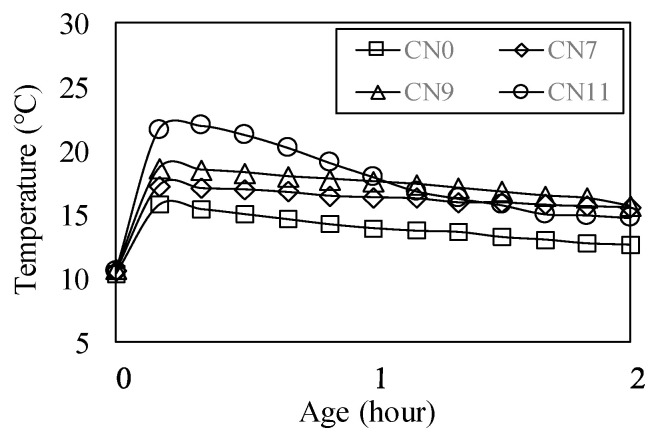
Temperature history (up to 2 h).

Figure 7 shows the results of the table flow test in each case. The flow value of CN0 was set to 186 mm, based on those of CN7, CN9, and CN100, which were decreased by 6.5, 9.2, and 25.9%, respectively. As the amount of CN was increased, the flow value tended to decrease. Figure 8 shows the history of the internal temperature of the mortar (~2 h). The temperature peaks were 15.2 °C for CN0, 17.1 for CN7, 18.6 for CN9, and 22.0 for CN11. As the amount of CN was increased, the temperature tended to increase. When CN was added, the NO_2_^−^ and NO_3_^−^ reacted rapidly with the C_3_A in the cement to produce nitrite/nitrate hydrate [13,15,17,18,22]. These results indicate that when a considerable amount of CN is added, hydration is promoted, the mortar temperature increases, and the flow value decreases.

### 3.2. Compressive Strength Properties

**Figure 9:**
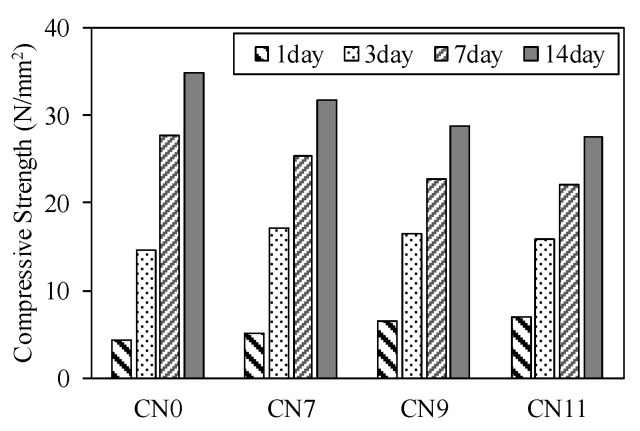
Compressive strength.

**Figure 10:**
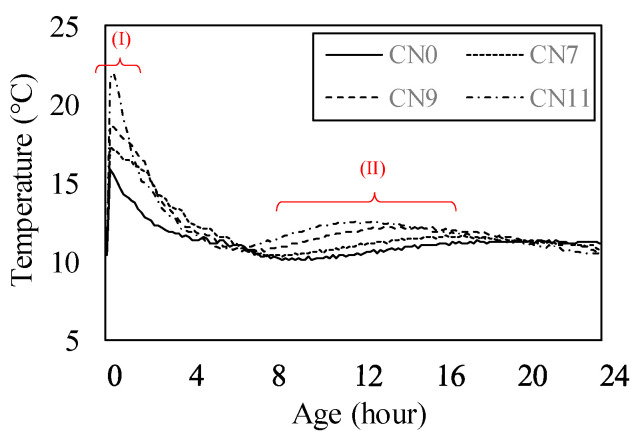
Temperature history (up to 24 h).

Figure 9 shows the compressive strength from 1 to 14 days for each case. The compressive strength on Day 1 was 4.38 N/mm^2^ for CN0, 5.15 for CN7, 6.51 for CN9, and 7.03 for CN11, and tended to increase with the amount of CN. Figure 10 shows the history of the mortar’s internal temperature (~24 h). In particular, the temperature peaks at 0–4 h and 6–18 h increased [16], and the time required to reach the peak decreased. With an increase in the amount of CN, the temperature tended to increase; moreover, the amounts of NO_2_^−^ and NO_3_^−^ increased and reacted rapidly with the C_3_A in the cement, which promoted hydration, increased the mortar temperature, and increased the amount of nitrite/nitrate hydrate. Therefore, the strength on Day 1 was considered to have increased. However, the compressive strength, by Day 3, tended to decrease as the amount of CN was increased. Furthermore, this tendency became remarkable after 7 days, and the strength in the case of CN addition was below that of CN0. With an increase in the amount of CN, the amounts of ettringite and nitrite/nitric acid hydrate produced also increased. Therefore, on Day 1, the structure became dense and the strength increased. After 3 days, when CN was added, a considerable amount of H_2_O was consumed, with an increase in the amount of nitrite/nitrate hydrate [15,17,18,22]. This relatively reduced the amounts of C-S-H and Ca(OH)_2_ produced by the reaction in ordinary cement, and made the structure without CN denser than that with CN. Therefore, the strength was considered to increase.

### 3.3. Void Structure Properties

**Figure 11:**
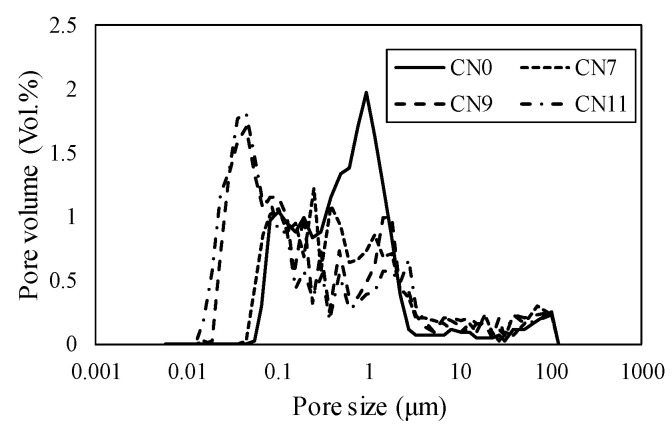
Void distribution (1 day).

**Figure 12:**
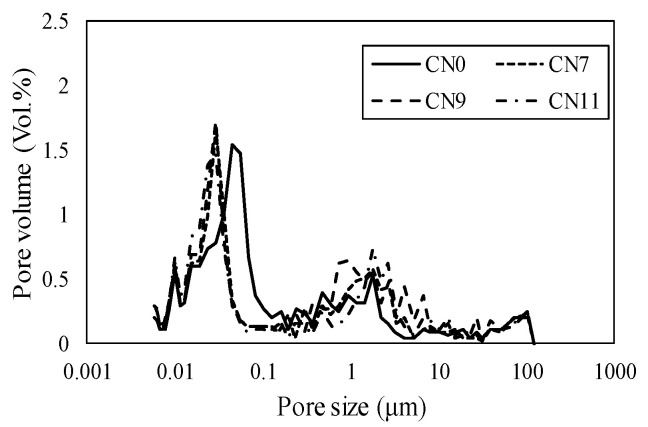
Void distribution (14 days).

Figure 11 shows the void distribution on Day 1, where CN1 had a void distribution in the range of 0.5 to5 μm, CN7 from 0.1 to 3 μm, CN9 from 0.05 to 0.5 μm, and CN11 from 0.03 to 0.1 μm. According to these results, the void diameter and volume tended to decrease as the amount of CN added was increased. In particular, in the cases of a considerable amount of CN being added (CN9 and CN11), many voids were formed within the range of 0.05 μm or less, which was considered to substantially affect dry shrinkage [16]. Figure 12 shows the void distribution by Day 14. In all cases, the void volume tended to be smaller than that on Day 1. These results indicate that CN addition increased the amount of nitrite/nitrate hydrate produced immediately after mixing, and promoted hydration; moreover, the voids were filled, resulting in a good strength on Day 1. On the other hand, after 14 days, there was no clear relationship between the void structure and the fact that the case of CN addition had a lower compressive strength than that with no addition of CN.

## 4. Shrinkage Properties of Mortar with CN

### 4.1. Restrained Drying Shrinkage

**Figure 13:**
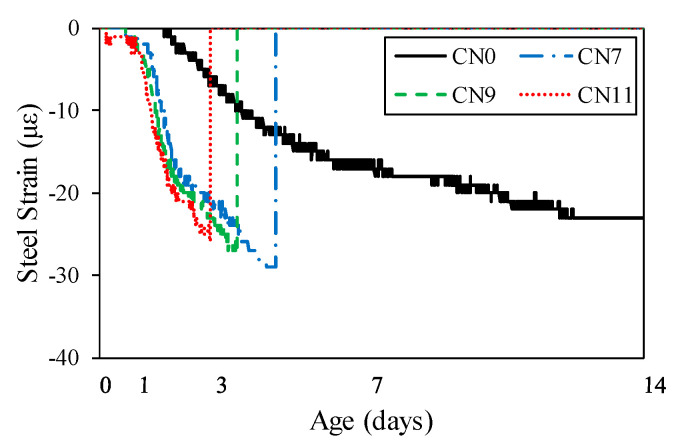
Restraint shrinkage.

Figure 13 shows the restrained shrinkage strain results obtained for the ring test. Table 5 shows the occurrence of cracks in each test piece and the number of days until the crack occurred. First, the shrinkage started after casting, after ~10 h for CN11, 11 h for CN9, 12 h for CN11, and 36 h for CN0. In particular, in the case of CN addition, shrinkage gradually occurred ~6 h after casting, after which cracks occurred in the process of increasing shrinkage. The cracking dates were 2.8 days for CN11, 3.6 days for CN9, and 4.4 days for CN7. The cracks tended to occur faster as the amount of CN was increased. The restrained shrinkage strains at the time of cracking were 25 μ for CN11, 27 μ for CN9, and 30 μ for CN7. On the other hand, in the case of CN0, no cracks occurred during the measurement period of this experiment.

### 4.2. Restrained Tensile Stress and Crack Potential

The restrained tensile stress can be calculated by Equation (1) using the radii of the concrete and steel ring, restrained shrinkage strain, and elastic modulus of the steel ring, assuming that the concrete poured into the ring specimen had a uniform shrinkage in the shear plane with a linear behavior [21,23,24,25].

(1) $$\sigma_{\theta imax}=\;\frac{\left(\gamma_{os}2-\gamma_{is}2\right)}{2\gamma_{os}2}\cdot \frac{\left(\gamma_{im}2+\gamma_{om}2\right)}{\left(\gamma_{om}2-\gamma_{im}2\right)}\cdot E_{st}\cdot \varepsilon_{st}$$

Here, *σ_θimax_* indicates the restrained tensile stress, *γ_is_* and *γ_os_* indicate the internal and external radii of the steel ring, *γ_ic_* and *γ_oc_* indicate the internal and external radii of the concrete, *E_st_* indicates the elastic modulus, and *ε_st_* indicates the restrained shrinkage strain.

**Figure 14:**
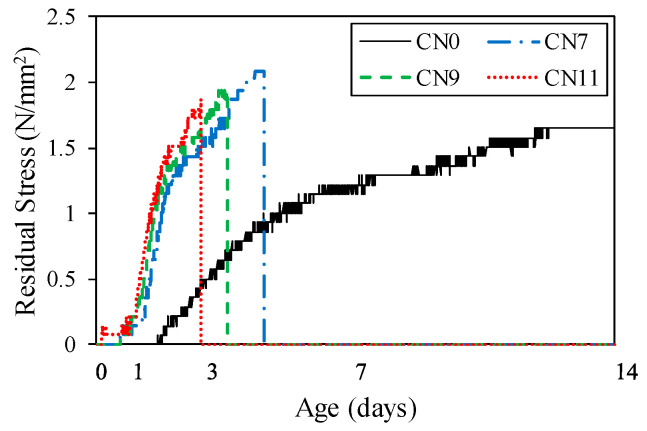
Restraint tensile stress.

**Figure 15:**
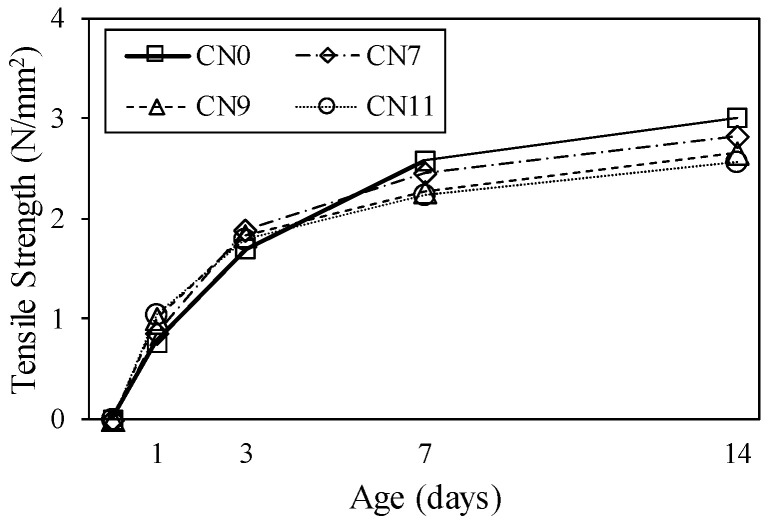
Tensile strength.

**Figure 16:**
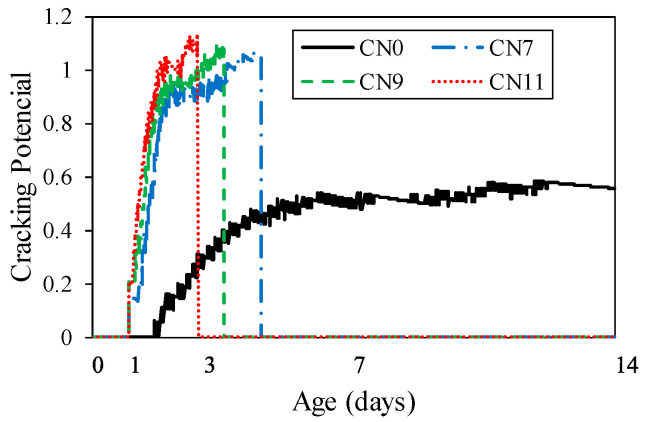
Cracking potential.

Figure 14 shows the restrained tensile stress calculated by Equation (1), which tended to increase with the restraint shrinkage strain. The maximum restrained tensile stress was 1.8 N/mm^2^ for CN11, 1.9 N/mm^2^ for CN9, and 2.1 N/mm^2^ for CN7, and cracking occurred after reaching the maximum tensile stress. The restrained tensile stress increased with the amount of CN added because of the increase in the stress generated in the inner steel ring. This was confirmed to accelerate the crack occurrence in the mortar. It is considered that the stress relaxation was reduced by the tensile creep. On the other hand, the cracking potential is calculated from the ratio of restrained tensile stress to tensile strength. Figure 15 shows the change in tensile strength over time, and Figure 16 shows the crack potential in each case. The tensile strength was calculated by Equation (2) using the result from the compressive strength [26]:

(2) $$\sigma_{B}=0.291\cdot Fc^{0.658}$$

Here, *σ_B_* refers to the tensile strength and *F_c_* refers to the compressive strength.

In the case of CN addition, the cracking potential increased between the ages of 1 and 2 days. The possibility of crack occurrence became very high at an early age, compared to the case of no CN addition. According to this result, under the restraint conditions in this test, with an increase in the amount of CN added, both the shrinkage and crack occurrence possibility increased.

### 4.3. Degree of Restraint

**Figure 17:**
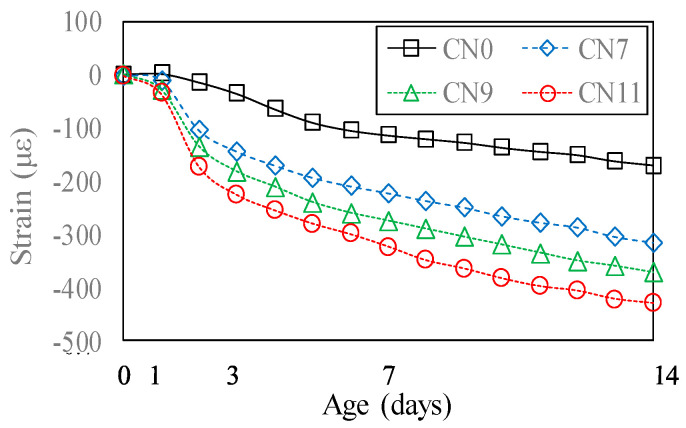
Unrestrained drying shrinkage.

Figure 17 shows the changes in the drying shrinkage strain over time. On Day 1, after the start of drying shrinkage, the drying shrinkage strain was 1.5 μ for CN0, 10.5 μ for CN7, 29 μ for CN9, and 36 μ for CN11, indicating that the drying shrinkage tended to increase with the amount of CN added. On Day 14, after the start of drying shrinkage, the drying shrinkage strain was 170 μ for CN0, 315 μ for CN7, 372 μ for CN9, and 430 μ for CN11, indicating that the drying shrinkage on Day 1 tended to increase with the amount of CN added, and became more pronounced. These results confirm that drying shrinkage increases with the amount of CN added, and agree with the restrained shrinkage results; moreover, when a considerable amount of CN was added, restrained shrinkage was considered to increase with drying shrinkage.

**Figure 18:**
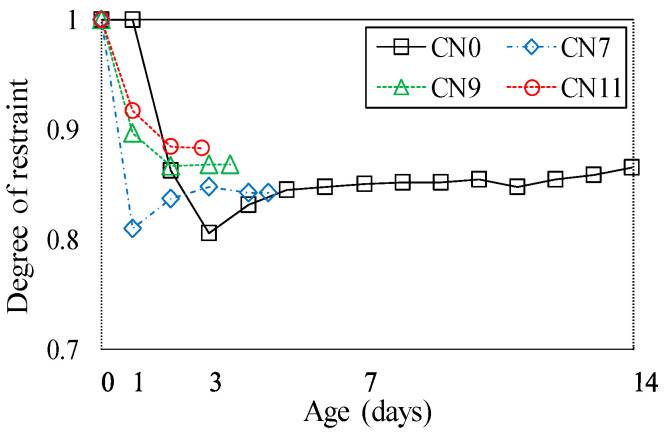
Degree of restraint.

Figure 18 shows the degree of restraint in the specimen by the restraining body which, in this test, was calculated using Equation (3), in order to evaluate the cracking [23,24,25].

(3) $$\varphi =1-\frac{\varepsilon_{st}\left(t\right)}{\varepsilon_{sh}\left(t\right)}$$

Here, *φ* represents the degree of restraint, *ε_st_* represents the amount of restrained shrinkage, and *ε_sh_* represents the amount of drying shrinkage. *ε_st_* is calculated by taking the concrete age of 0 days as the start of the restrained shrinkage.

The degree of restraint tended to increase slightly with the amount of CN added, and was ~0.84 for CN7, ~0.87 for CN9, and ~0.88 for CN11. This confirmed that the time the cracking occurred, caused by the degree of restraint, became earlier as the amount of CN added was increased. Meanwhile, in the specimen with no CN added, after decreasing to 0.80, the degree of restraint tended to gradually increase, reaching ~0.86 on Day 14. The results showed that when a considerable amount of CN was added, the cracking potential reached a maximum, and cracking occurred when the degree of restraint was between ~0.84 and 0.88. Therefore, it is considered necessary to examine the crack occurrence under the restraint conditions when using CN.

## 5. Conclusions

In this study, each experiment was conducted in order to clarify the shrinkage behavior and cracking of mortar when the amount of nitrite-based accelerator, used as an anti-freezing agent, was changed. The results are summarized as follows:

(1) When the amount of CN was increased, hydration accelerated, the mortar temperature increased immediately after casting, and the fluidity decreased.
(2) On Day 1, the addition of a considerable amount of CN promoted hydration and formed a large amount of nitrite/nitrite hydrate, resulting in dense voids and increased strength.
(3) As the amount of CN was increased, shrinkage increased and its start time became earlier.
(4) Under the restraint conditions in this study—from the results of the cracking potential and degree of restraint—as the amount of CN was increased, the shrinkage and crack occurrence possibility increased.

## Figures and Tables

**Table 1:** Properties of the materials used (Data from [18]).

Materials (Code)	Properties
Cement (C)	Normal Portland cement, density: 3.16 g/cm^3^ (Taiheiyo Cement, Tokyo, Japan)
Fine aggregate (S)	No. 5 silica sand, absolute dry density: 2.61 g/cm^3^, Water absorption: 0.26%, fineness modulus: 2.16 (Tochu, Tokyo, Japan)
Anti-freezing agent (CN)	Nitrite nitrate-based accelerator = calcium nitrite (Ca(NO_2_)_2_); calcium nitrate (Ca(NO_3_)_2_) (Nissan Chemical, Tokyo, Japan)

Note: CN: Anti-freezing agent = nitrite + nitrate-based accelerator (Ca(NO_2_)_2_ + Ca(NO_3_)_2_).

**Table 2:** Properties of the anti-freezing agent (Data from [18]).

Code	Component	Component Ratio	pH	Specific Gravity
CN	Ca(NO_2_)_2_	23.02%	9.3	1.43 g/cm^3^
	Ca(NO_3_)_2_	22.81%		

**Table 3:** Proportions of the mortar mix (Data from [18]).

Type	W/C (%)	S/C	Unit Content (kg/m^3^)			Anti-Freezing Agent (C × %)
			W	C	S	CN
CN0	50	2.5	281	562	1407	0
CN7						7
CN9						9
CN11						11

Note: W/C: Water–cement ratio; S/C: Sand–cement ratio; CN0: Mixing amount of anti-freezing agent = 0%; CN7: Mixing amount of anti-freezing agent = 7%; CN9: Mixing amount of anti-freezing agent = 9%; CN11: Mixing amount of anti-freezing agent = 11%.

**Table 4:** Experimental conditions and evaluation method (Data from [18]).

Temperature Condition	Experimental Period	Subject and Method of Evaluation	
		Physical Properties	Shrinkage Properties
10 °C	Casting—14 days	Flow test	Un/Restrained shrinkage
		Compressive strength	Tensile strength
		Temperature history	Crack potential
		MIP	Degree of restraint

Note: Physical and shrinkage properties: All cases; flow test: Immediately after placement; temperature history: Casting—14 days.

**Table 5:** Crack configuration and cracking days.

Cracking	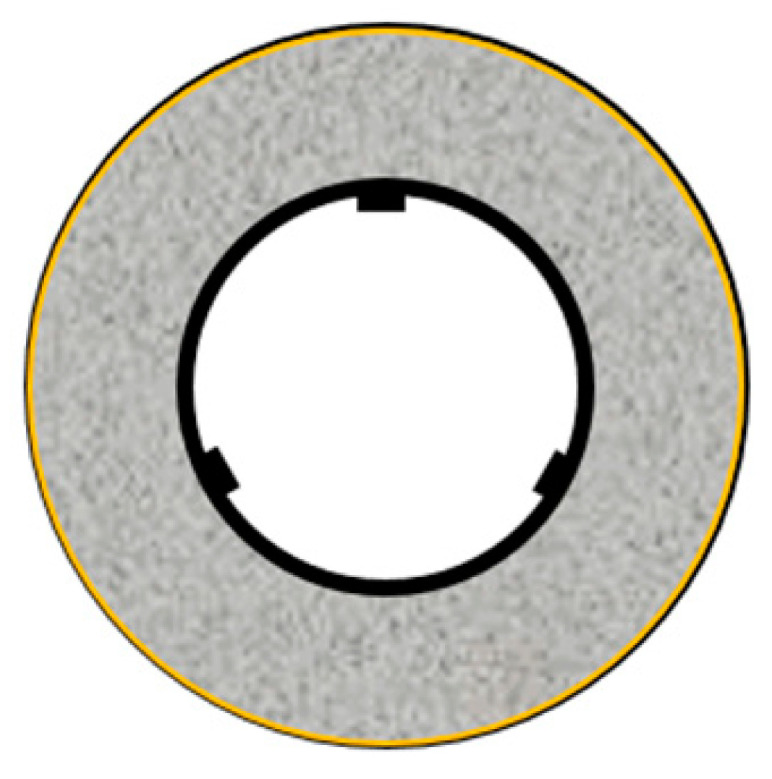	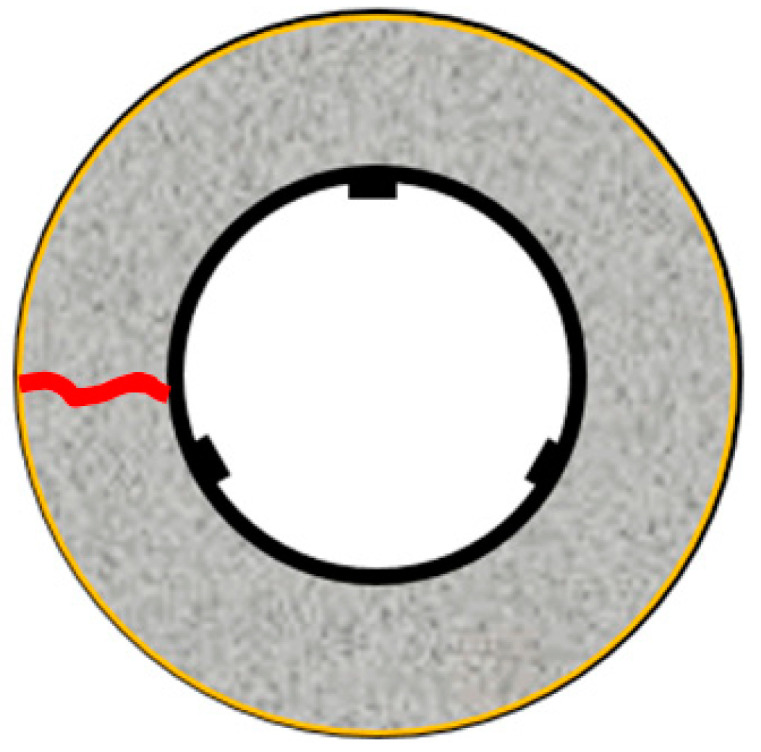	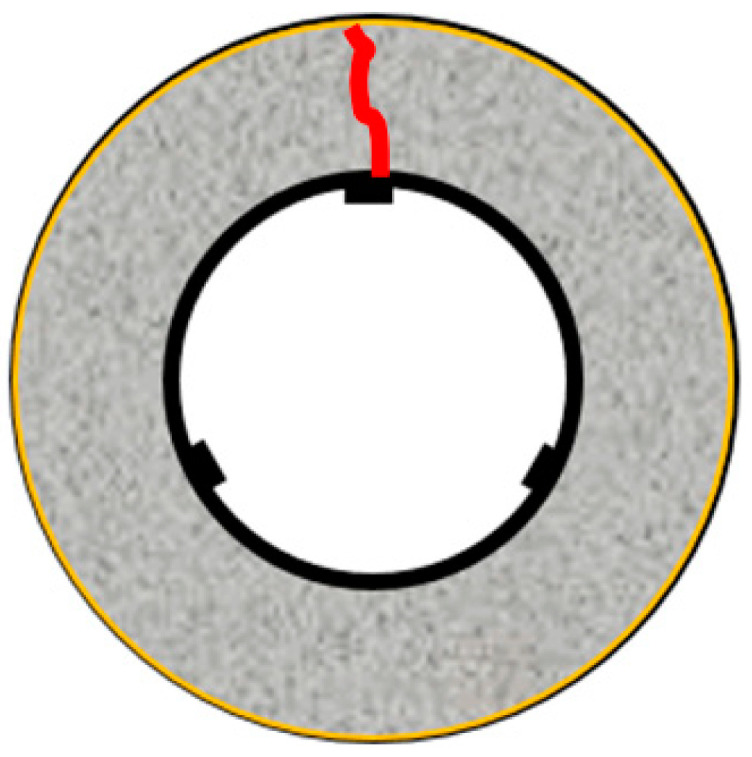	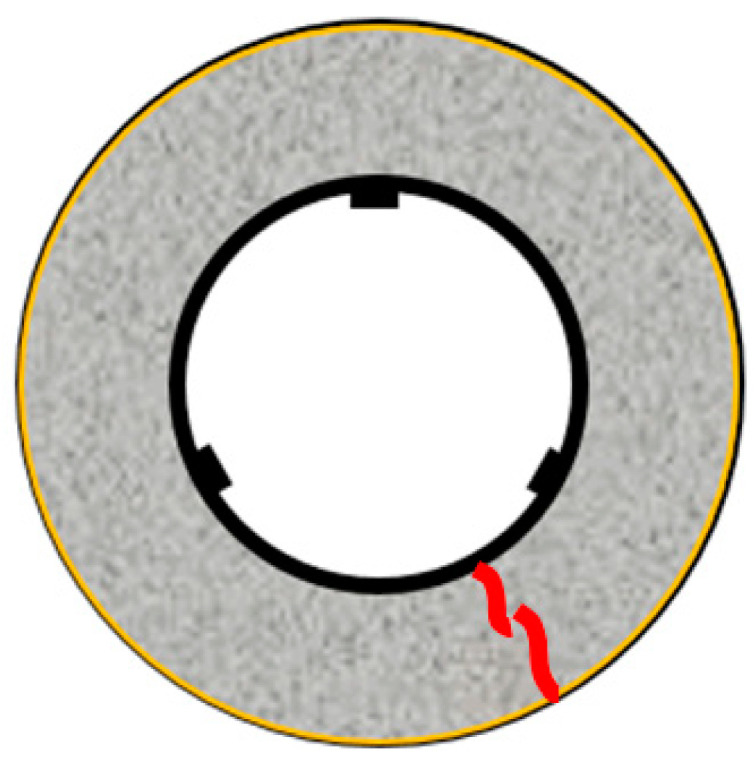
Case	CN0	CN7	CN9	CN11
Days of Cracking	-	4.4 days	3.6 days	2.8 days
